# UK homecare providers’ views about, and experiences of, digitalisation: A national survey

**DOI:** 10.1177/20552076241255477

**Published:** 2024-05-22

**Authors:** Jan Healey, Vanessa Davey, Jennifer Liddle, Gareth O’Rourke, Barbara Hanratty, Bryony Beresford

**Affiliations:** 1 Social Policy Research Unit, School for Business and Society, 8748 University of York, York, UK; 2 Population Health Sciences Institute, Newcastle University, Newcastle upon Tyne, UK

**Keywords:** Social care, homecare, digitalisation, digital transformation, domiciliary care‌

## Abstract

**Objective:**

Using digital systems to support the management and delivery of social care is a priority for UK governments. This study explored progress towards, and experiences of, digitalisation in the homecare sector and providers’ views on contributing client data to a national policy/research dataset.

**Methods:**

Over 150 UK homecare providers completed an on-line survey (October–December 2022). The survey was hosted on *Qualtrics* and comprised fixed- and free-text response questions. The recruited sample aligned with the profile of UK homecare providers in terms of use of digital systems, organisation type and size.

**Results:**

Almost all respondents (95.5%) were using digital systems, in part or exclusively, to support care delivery. However, many (42.7%) reported a desire to further digitalise or a dissatisfaction with existing systems. Findings highlight the time and work involved in choosing a a software system, with the decision regarded as relatively high risk. Over 50 different software systems were being used across the sample. Most respondents (72.5%) supported the creation of a national dataset on homecare users. However, support and recompense are likely to needed to secure buy-in from what is a predominantly private sector context.

**Conclusions:**

Findings suggest a complex and changing situation, with numerous different digital systems being used and the sector at different stages of digitalisation. The high-pressure, low margin context of UK homecare appeared to be exerting an influence on progress towards digitalisation. Evaluations of government strategies to stimulate and support digitalisation in this diverse and predominantly private sector context will be valuable.

## Introduction

### Background and rationale

In 2021, the English government's Department of Health and Social Care set out a 10-year plan to reform social care, with similar programmes of work underway in other UK countries.^1–3^ It has three core objectives: (i) people have choice, control and support to live independent lives; (ii) people can access outstanding quality and tailored care and support; and (iii) people find adult social care fair and accessible.^4^ The digital transformation of social care is a core strategy to achieving this plan, with over £150 million investment planned to support and increase the use of digital technologies to innovate approaches to care, and to ensure digital systems can replace manual or physical systems and processes (e.g., paper-based records, manual rostering processes). Here, digital systems are presented as both supporting the quality of care (e.g., outcomes, safety) and care efficiency, though this is relatively untested.^5^

A number of key work programmes and targets have been developed to progress the digital transformation of social care in England. These include the creation of a ‘minimum operational dataset’ (MODS) which specifies and defines the core information which a digital social care record must include. Crucially, the MODS draws on international e-health data standards^6,7^ to ensure interoperability between digital systems being used by health and social care providers. Whilst the software system used is not mandated, software systems which adhere to MODS and data security requirements can apply to listed by government as an assured software system supplier.^8^ In addition, the aspiration is that, by March 2024, the majority of registered social care providers will be using digital care records. In the longer term, digitalisation is regarded as enabling new national collections of social care data with the potential for data linkage with health datasets.^9^ Alongside this, there is a commitment to ease and improve researchers’ access to such datasets thus supporting the building of a substantive evidence base on social care.^9^

Achieving digitalisation of UK homecare comes with a number of challenges.^10^ First is the lack of digital readiness and digital maturity within the sector.^11^ A 2021 survey of digital readiness in social care found just a fifth of social care providers regarded themselves as ‘digitally expert’, though self-defined digital ‘novices’ had decreased compared to a previous survey in 2019 (14% vs. 24%).^12^ The survey also found evidence of concerning levels of inadequate policies or practices with respect to data protection and security. Likewise, recent government estimates are that just around half of social care providers have digital care records.^9^ A second challenge to digitisation is the number and variety of homecare providers. In 2021/22 in England, for example, there were 10,800 homecare providers^13^ ranging from services operating out of a single office (or operating base) through to large national chains with multiple local offices or branches. For providers with a small management and administrative team, the substantial work associated with digitisation may well present challenges in terms of capacity, skills and financial cost. Third, and unlike the UK health care system, almost all homecare providers are private, for-profit businesses or, less commonly, not-for-profit rather than public sector run services.^14^ Thus, business risk becomes a salient concept and, for owners of smaller companies, the potential for personal financial risk is inherent.^15^ The precarious nature of the social care sector (e.g., small-sized businesses, high staff turnover, insufficient public sector funding, narrow business margins) in the UK adds to this.^16^ Finally, and no less important, are the multiple, and sometimes unpredictable, demands and responsibilities associated with running a homecare service which can threaten the time available to plan for and implement digital transformation within the service.

### About the study

To date, there has been little independent research into digital readiness in homecare and homecare providers’ attitudes, concerns and experiences of digitisation,^17^ partly reflecting low levels of research on homecare more generally.^18^ This paper reports findings from a survey of UK homecare providers (i.e., owners, managers) that collected data on: current information management systems and types of data collected; any plans to move to digital systems or change or improve existing digital systems, including perceived barriers to change; and their views on the possibility of a national dataset of homecare users for policy-making, commissioning and research purposes to which homecare providers would voluntarily contribute anonymised data. The survey also collected detailed data on the types of data routinely collected and the format in which they are recorded and stored. Findings on these topics are reported elsewhere.^19^ This survey was one component of a study on data and digitisation in homecare (the DACHA-DOM study) which itself is nested in a programme of work on data and digitisation in care homes.

## Methods

### Study aims

The aim of the study was to describe the information management systems being used by UK homecare providers, their experiences of, or intentions, to move to digital systems, and their views about the possibility of contributing to a national dataset of homecare users.

### Study design

This was an exploratory study comprising a cross-sectional survey of UK homecare providers which collected quantitative and qualitative data. The study design was pragmatic and the survey used a convenience sampling approach.

#### Study population

Inclusion criteria were

- Respondent is the owner or manager of an organisation providing homecare where:
  - at least some of the care was in the form of regular home visits,
  - the care provided was regulated by the Care Quality Commission.

Survey instructions requested that it was completed by the owner/director or a manager.

### The survey and survey development

An on-line survey was used, hosted on the Qualtrics survey platform (Qualtrics, Provo, UT, USA). It comprised 45 questions which were predominantly fixed-response and was designed to be completed anonymously, but with the option for respondents to state the homecare organisation they were representing.

The survey was organised into six sections, with this paper reporting on data collected by the following four sections: the type, size and funding status of homecare organisations; the systems used to collect and store information; satisfaction with current information management systems and plans for changes or improvements; and views about contributing to a hypothetical national dataset on people who use homecare.

Drafts of the survey were reviewed at two stages of survey development by academics independent of the research team but also researching the digitisation of social care, and senior representatives of two national organisations representing UK homecare providers. A near-final version was then piloted with senior managers of two homecare providers using cognitive interview techniques^20^ with revisions made as a result of the first pilot tested with the second pilot. A copy of the survey questions is provided in Supplementary File #1.

### Data collection

An email invitation to take part in the survey (including anonymous hyperlink to the survey) was distributed to members of the UK's two national bodies for homecare providers. The combined membership of these bodies is over 6500. Together they represent for-profit and not-for-profit, and small and large, homecare providers. At the same time, a short article about the survey was published in one of the UK's most popular ‘trade magazines’ for homecare owners and managers. The research team also distributed the email invitation via their existing networks and posted information about the survey on Twitter. One email reminder was sent. The online survey was operational between 19 October 2022 and 9 December 2022. The study information sheet (including data privacy notice) was attached to the email invitation. As this was an anonymous survey, consent was not recorded. Survey submission was assumed to indicate a willingness to take part in study on the basis of information provided in the invitation and study information sheet.

### Data analysis

The data file was downloaded from Qualtrics into Excel. Where respondents had chosen to provide the name of their homecare organisation (this was optional), the dataset was checked for duplicate responses. In the small number of instance where this was identified, the more complete response retained. Where the name of the homecare organisation was not provided, responses in the same region, business type and size were examined to identify potential instances of duplicate responses. None were identified. After data cleaning, quantitative data was imported into SPSS (SPSS 25.0) for descriptive analysis (e.g., frequency counts, crosstabs). Data generated from questions which used a ‘free text’ response format was analysed using conventional content analysis.^21^

Information provided by survey respondents on the software packages/systems they used was analysed separately, with scrutiny of software product websites used to determine the types of functions offered. These were categorised as follows: roster/scheduling; care assessment, planning and reviews; care delivery/care records; administration of medication; visit logging; recording of safeguarding concerns/alerts; mobile app for homecare workers, mobile app for family members; client/user satisfaction surveys; care worker travel; staff training & supervision log; finance (e.g., invoicing, accounting); other business management functions. Also recorded was whether the software was generic or homecare specific, and where relevant, if the software systems was registered as a government-assured digital social care record supplier. This information was recorded on an Excel spreadsheet.

## Results

### Sample

The final survey sample comprised 155 respondents following removal of duplicate (*n* = 5), incomplete (*n* = 28) or ineligible (*n* = 9) responses. Respondents included business owners/franchisees (*n* = 59, 38.0%), executive or other directors (*n* = 28, 18.1%) and registered/senior managers (*n* = 64, 41.3%). Four respondents held a different role (management or administrative) within their organisation.

The types of homecare organisation represented in the sample included local independent businesses, care/homecare chains and owners of a homecare franchise, third sector/charity providers and a small number of local authority in-house homecare services, see Table 1. The great majority were for-profit businesses. Almost three quarters of respondents had a single operating base but a small number (*n* = 17) were reporting on a multi-region or national homecare chain. This diversity of homecare provider type is reflected in the wide range of caseload size. In addition to regular domiciliary care visits, just over a third of sample were also providing reablement (a short-term intervention delivered to people living in their own homes which seeks to restore, or maximise, independence in activities of daily living), and a similar proportion were providing live-in care. A minority of the sample provided other types of social care, either residential care and/or extra care housing/assisted living facilities. Just under half of respondents had caseloads which were all or mostly funded by one or more local authorities, just over a third reported they predominantly served the self-funded market, and a fifth reported their caseload included around equal numbers of local authority and self-funded clients. Finally, six out of ten respondents said their caseloads could include homecare funded by the NHS via continuing health care (CHC) budgets.

**Table 1.** Characteristics of the homecare organisations represented in the survey.

Nature of organisation (*n* = 155/155)		
Local independent business	93	60.0
Franchise	30	19.4
Chain	17	11.0
Third sector/charity	10	6.5
Local authority in-house service	5	3.1
Business type (*n* = 154/155)		
For profit	133	86.4^a^
Not for profit	16	10.4^a^
Public sector	5	3.2^a^
Number of operating bases (offices, branches, franchises) (*n* = 142/150^b^)		
1	106	74.7
2–3	21	14.8
4–10	8	5.6
11–19	2	1.4
20+	5	3.5
Caseload^c^ (*n* = 151/154)		
30 or fewer	38	25.2
31–75	39	25.8
76–180	37	24.5
181–26,000^d^	37	24.5
Homecare services provided in addition to regular domiciliary care visits ((*n* = 151/155)		
Live-in care	54	35.8
Reablement	52	34.4
Not also providing live-in care or reablement	45	29.8
Types of social care provided (*n* = 155/155)		
Homecare only	121	78.1
Home care + Care home	16	10.3
Homecare + Extra care housing / assisted living	18	11.6
How care is funded (*n* = 155/155)		
All or mostly local authority (LA) funded	70	45.2
All or mostly self-funded	53	34.2
Roughly equal numbers LA- and self-funded	32	20.6
Caseload can include homecare commissioned by continuing health care (CHC) budgets (*n* = 155/155)		
Yes	94	60.6
No	61	39.4

^a^ Equivalent national data^14^: 85%, 12%, 3%.

^b^ Excludes in-house local authority homecare services.

^c^ Caseload for homecare service only where also provide other type(s) of social care.

^d^ Caseload size 181–249: *n* = 6 (4.0% of total sample); caseload size 250–499: *n* = 16 (10.6%); caseload size 500–899: *n* = 6 (4/0%); caseload size 900–4900: *n* = 5 (3.3%); caseload size 5000+: *n* = 4 (2.6%).

### Information management systems and client data held in digital format

Around half of survey respondents (49.7%) reported their information management systems were ‘all or predominantly’ digital with just a small minority (4.5%) reporting using all or predominantly paper-based systems, see Table 2. The remainder reported using a mix of paper and digital systems.

**Table 2.** Nature of information system by number of offices/operating bases (*n* = 155).

Nature of information system	Number of operating bases/offices (*n*)					Total
	1	2–3	4–10	11–19	20+	
All or predominantly paper based	7	0	0	0	0	7
Mix of paper & digital	59	6	2	1	3	70
Predominantly digital	56	12	6	1	2	77
Total	122	18	8	2	5	155

Respondents with paper-based information systems were all homecare providers with a single operating base/office and caseloads ranging between 21 and 150 clients. Rate of use of all/predominantly digital systems (as opposed to mix of paper and digital) did not differ between providers with 2–3 operating bases (*n* = 18) and those with 4 or more operating bases (*n* = 15) (chi-square(3, 33) = 0.16, *p* = 0.691).

The survey asked about the types of client information held in digital format. (This could be either because data was collected/recorded using a digital system or digitised from paper records.) Around one in ten respondents did not store information about the care package or the care needs assessment in digital format, see Table 3. Digital information about clients and their care was mostly likely to be used to inform staffing/workforce planning and understanding or monitoring client needs, see Table 3. Fewer than three quarters of respondents used digital data to monitor client outcomes or client experience/satisfaction. Just over half used digital data to inform business decisions.

**Table 3.** Nature of information system and types and use of information available in digital format.

	*N*	%
Nature of information system (*n* = 155)		
All or predominantly digital files/software	77	49.7
Mix of paper records and digital files/software	71	45.8
All or pre-dominantly paper-based	7	4.5
Type of client information routinely available in digital format^a^ (*n* = 154/155)		
Basic information about the client (e.g., age, gender)	145	94.1
Record of daily care visits	141	91.5
Care package details	139	90.2
Care needs assessment information	135	87.7
Client satisfaction surveys	91	59.0
Use of digital information^a^ (*n* = 154/155)		
Inform staffing/workforce planning	126	81.8
Understand/monitor needs of clients	122	79.2
Identify staff training needs	116	75.3
Monitor client outcomes	105	68.2
Monitor client experience/satisfaction	105	68.2
Inform business development decisions	85	55.2

^a^ Survey question multi-response.

### Software systems being used

Across the survey sample, a total of 54 different software systems were being used (this excludes generic software/applications, e.g., Microsoft 365; Google). The majority (62.4%) reported using one system, over a quarter (28.4%) were using two systems, and almost one in ten (9.2%) three or more different systems. The functions offered by these systems included guiding and recording the care/needs assessment process, care package details, specifying and recording of care provided during a homecare visit, scheduling/rostering, workforce/HR, invoicing and/or payroll functions. Some systems offered all these functions, others were limited to one or two functions.

Nearly three quarters of respondents (*n* = 112) were using a multi-function care management software system (i.e., functionality includes: care needs assessment, planning and reviews, recording of care visits (including medication administration), rostering and mobile app for homecare workers, invoicing etc.). Across the sample, 20 different care management systems were reported, 10 of which were homecare-specific. These were being used by just under half (*n* = 48/112 (42.9%)) of those with a care management software system. At the time of writing, one third (*n* = 38/112) of respondents were using care management software system not currently listed on the government's register of assured providers.

Over four out of ten respondents (*n* = 47/112) were using the same care management software system, see Table 4. A further four systems (all homecare specific) were being used by a total of 36 respondents. At the time of writing, only one was registered as an assured digital social care record supplier. The remaining care management software systems (*n* = 15) were being used by 1–3 survey respondents.

**Table 4.** Relative use of digital care management systems across the survey sample.

	No. respondents using system	Registered as a digital social care record assured supplier	Homecare specific software?
Digital care management system ‘A’	47	Yes	No
Digital care management system ‘B’	14	No	Yes
Digital care management system ‘C’	11	Yes	Yes
Digital care management system ‘D’	6	No	Yes
Digital care management system ‘E’	5	No	Yes

Number of other care management software systems being used by 1–3 respondents = 15

### Provision of mobile devices to homecare staff

Just over a quarter of survey respondents (*n* = 44/154; 28.6%) provided all their homecare workers with a mobile phone. (Note: that the survey did not ask if homecare workers used their own phones to interface with digital care management systems (e.g., via an app)). Almost all reported mobile phones were supplied to support communication with office staff (*n* = 40) and scheduling visits (*n* = 39). Many were also using the devices to support communication between homecare staff (*n* = 35), completing client care records (*n* = 33) and/or time-tracking (*n* = 32). When asked if mobile connectivity impacted how they used mobile devices, 17/44 (31.8%) respondents reported this was the case. Poor connectivity was almost always attributed to rurality, though patchy signal coverage within buildings was also reported. Issues with connectivity were reported as impacting homecare workers access to the digital care records and real-time updating.

### Satisfaction with current information management systems

The survey asked respondents if there were things they would like to change or improve about their current information management system. Follow-up questions (using ‘free-text’ response format) collected data on reasons for and barriers to change. Over four out of ten respondents (*n* = 64/150; 42.7%) said they wanted to make changes or improvements, including over a third of those with all or predominantly digital systems, see Table 5. Not all those with all/pre-dominantly paper-based systems reported wanting to change their current system.

**Table 5.** Wish to change or improve current information management system by nature of current information management system.

Nature of information system	Wish to change or improve information management system		
	Yes	No	Total
All or pre-dominantly paper-based	5	2	7
Mix of paper records and digital files/software	33	36	69
All or pre-dominantly digital files/software	26	48	74
Total	64	86	150

Among those wanting to change or improve an existing digital/paper, or entirely digital, system, some wanted to reduce the number of systems they were using, or to reduce duplication across or within systems. Others reported being dissatisfied with the care management software system they were using. Reasons for this included: (a) software not having all the functionality they needed (e.g., lack of client experience or outcomes measurement, unable to record all the information required); (b) system inflexibility and unable to support person-centred assessment and care delivery; (c) system unreliability; or (d) the system was difficult to use (e.g., non-intuitive). Some said they wanted to change or improve how they used the digital data they held. This was described both in terms of streamlining reporting and care compliance processes and exploiting the potential to use data to predict increased risk of unplanned hospital admissions or other health/care risks. Finally, a couple of respondents reported wanting to improve the information homecare workers were entering into the system. For example, one respondent described observing a decline in the quality of information being recorded by their homecare workers that, had in some cases, reduced to ticking off a checklist and failing to add any further details as ‘free text’.

Financial costs and staff resistance to change were cited as barriers to implementing changes or improvements to information management systems, as was a lack of digital skills or confidence among staff. A lack of time was frequently reported as a barrier to change, including time needed to research the market, transfer paper or digital information into a new system, system implementation whilst maintaining care provision, and staff training. Some noted that the daily pressures of running a homecare business made it very difficult, if not impossible, to make time for digital changes or improvements.

The recruitment and retention challenges in social care mean that too much effort is having to go into shoring up fragile operations rather than in forward thinking and development – implementing new processes/systems requires people to attend to them. (S080)

A further barrier to increased digitisation identified by a few respondents was the fact that other services (e.g., district nurses, GPs) going into the home required access to, or used, their paper care record.

For those wishing to change software provider, a number of specific barriers were reported. These included concerns about whether or how data on the existing system could be accessed or transferred, the costs of paying for two systems whilst transferring or for the duration of the notice period, and uncertainties about whether making such a change would be beneficial or the software would deliver everything it promised.

It is extremely difficult to switch systems. Finding a new system is always a challenge – the sales team will tell you what the system does well, but you also need to try to figure out what it does badly! Once you have found a new system it can be difficult to leave the old system – our current company has a 12-month tie in that requires 3 months advanced notice. And then there is the issue of maintaining access to all the data held on the previous system – this is a huge problem. (S140)

Some reported that, whilst dissatisfied with their current care management system, they had been unable to find a system which offered everything they needed. A number of responses conveyed the weight and significance of decisions about changing to a new care management system, and the risks it brought as the following response illustrates.

There are many IT care software available but unsure which one of these is able to record everything we need. We are wary of changing from our current system as this would cause so much disruption to daily running of the business. (S025)

A lack of digital awareness among clients or family members was also cited as a barrier to increasing the digitisation of care management processes. Finally, some referred to a lack of control over the digital system they used, either because of requirements or conditions imposed by local authority commissioners or because it was determined by the franchisor.

### Views about contributing to a ‘National Homecare Dataset’

Respondents were asked if, in order to improve understanding of the population of older people using homecare, a national ‘dataset’ of people using homecare populated by non-identifiable data voluntarily submitted by homecare providers was a good idea. Almost three quarters agreed, including all those whose information management system was all/predominantly paper-based, see Table 6. The great majority of the remainder said they were unsure rather than being unsupportive.

**Table 6.** Views about a ‘national homecare dataset’ by nature of current information management system (*n* = 142/155).

Nature of information management system	Good idea? (*n*)			
	Yes	No	Unsure	Total
All or pre-dominantly paper-based	7	0	0	7
Mix of paper records and digital files/software	39	3	22	64
All or pre-dominantly digital files/software	57	2	12	71
Total	103 (72.5%)	5 (3.6%)	34 (23.9%)	142

When asked if they would consider contributing to such a dataset, just over a third said they would consider this (see Table 7). The majority of the remainder stated that they would possibly consider contributing to it (*n* = 76/92; 82.6%).

**Table 7.** Views on whether would consider contributing to a ‘national homecare dataset’ (*n* = 141/155).

Would your homecare service/organisation consider contributing to such a dataset?	*n*	%
Yes	49	34.75
Maybe	76	53.09
No	3	2.12
Don’t know	13	9.21

Respondents were asked to report what might affect their willingness or ability to submit data to such a dataset from a list of nine possible reasons presented to them, see Table 8. The costs to their organisation of devoting staff time to this activity, concerns about data privacy and client willingness were the most frequently identified barriers to contributing to a dataset. Software and hardware costs were the next most frequently identified barriers and just under a third believed commercial sensitivities might be a barrier. Around a fifth questioned the ease at which it would be possible to extract required data from their information management systems. Among ‘other’ reasons entered, lack of staff time was the main issue, with some noting the time already devoted to supplying data to external agencies (e.g., Care Quality Commission, local authorities, Adult Social Care Workforce dataset). Being unclear of the benefits to themselves was also identified as a further reason why homecare providers may be unwilling or unable to contribute to a national homecare dataset.

**Table 8.** Factors affecting willingness or ability to submit data to a national homecare dataset (*n* = 138/155).

	*n*	%
Staff time costs	114	75.4
Concerns around clients’ privacy/GDPR	94	68.1
Client willingness for their anonymised data to be shared	94	68.1
Software costs	69	50.0
Hardware costs (e.g., mobile devices, computers)	48	34.8
Commercial sensitivities	42	30.4
Insufficient data management/tech skills within management/admin team	32	23.2
Data stored in multiple formats/places	29	21.1
Some/all data stored in paper format	26	18.8
Other	9	6.5

## Discussion

This study sought to explore current approaches to information management being used by homecare providers, satisfaction with existing systems and plans for change, including the perceived barriers to change. It also canvassed opinion on the notion of a national dataset of homecare users populated by anonymised data submitted by homecare providers, and the factors that may affect willingness or ability to contribute to such a dataset.

This was an exploratory study and adopted a pragmatic approach with convenience sampling used to recruit respondents. Findings therefore cannot be taken as representative of the entire population of UK homecare providers. Invitations to complete the survey were distributed by two national bodies which together represent the different types of homecare providers operating in the UK and with a combined membership of over 6500 providers. The response rate was disappointing, likely reflecting multiple and pressing demands on owners/managers’ time, and perhaps also an unfamiliarity with research, either as a user of research evidence or as a research participant.^18^ However, the achieved sample had a similar profile to national homecare providers in terms of business type, digitalisation and the proportion of public sector-funded and self-funded clients.^22^ It also represents the wide range of homecare provider organisation sizes, with small, single operating base businesses represented as well as some of the main UK homecare chains.^22^ As such, the study offers novel evidence relevant to informing and supporting the success of UK social care digital transformation programmes. More widely, it provides insight into the relatively neglected perspective of homecare company owners and senior directors/managers regarding planning for and implementing digital systems^5,17^ with most existing work in this area focussing on the impacts of digitalisation on care workers or homecare clients and their families.

Almost all respondents reported their information management systems were entirely digital or comprised a mix of digital and paper records. Many different software systems or packages were being used, offering varied functionality. Around a third of the sample were using multiple systems, perhaps reflecting how homecare providers may digitise one aspect of care delivery (e.g., rostering, check-in/check-out monitoring) and later decide to digitise another component (e.g., care planning, care records). Having multiple software systems was a common reason for dissatisfaction with existing information management systems: an issue we return to later.

Three quarters of our sample were using a multi-function care management software system with numerous different systems represented, perhaps reflecting the increased interest in the sector by software developers. There was, however, one dominant software provider, with four other systems also being used by multiple respondents. Half of the systems were designed specifically for homecare, raising questions about the feasibility of seamless digital transfers should homecare clients move into residential care.^23^ Furthermore, a third of respondents were not using a software provider registered as an assured supplier by the government's Department for Health and Social Care. This raises several issues. First, it is possible that some non-registered systems do not support recording of data as specified by the government's new MODS for social care, or they may not offer the same level of data security.^24^ Furthermore, the longer-term viability of non-registered software providers must also be questioned, raising the possibility that some homecare providers may unexpectedly face having to finance and implement new systems.

A sizeable minority of survey respondents reported they wanted to change or improve the digital system(s) they were currently using. This is to be expected in a context where digitalisation is still in process.^25^ There were two key reasons for wanting to change existing systems. First, a desire to move from multiple to a single system. Second, their existing system not offering the functionality or flexibility required. However, there was also evidence of homecare providers having to tolerate unsatisfactory systems because of a lack of time and financial resources or, alternatively, the lack of a better option. Such findings align with previous work on this topic.^5^ Free-text responses conveyed the time investment required to research, select and move to (or switch) care management software: something which could be hard to protect given the pressing and unpredictable nature of many of the demands on senior managers’ time. Since the study was completed the government have published guidance for homecare providers on selecting a software system.^26^ This may go some way to meeting the information and guidance needs of homecare providers. This is, as yet, untested. Alongside this were the descriptions of such decisions carrying high financial stakes, both in terms of uncertainty about the ‘return on investment’ and the risk of ‘business disruption’ and the direct impacts this would have on their clients. Whilst financial support from government is available to support digitalisation,^27,28^ the degree to which this covers the true costs of selecting and implementing a digital system is unknown. Furthermore, such support excludes those wanting to *change* the digital system they use.^27,28^ Importantly, taken alongside other research,^9^ our survey findings suggest this group may be much larger than those moving from paper-based to digital records.

With respect to wanting to make changes in how existing digital systems were used, a number of different types of change were articulated. They included a desire to better utilise the data held. The wider literature suggests this is not uncommon^25^ and points to the investment needed in analytical capabilities within an organisation. To date, the support and training on this which is freely available from the government's digital transformation programme appears limited,^29^ leaving homecare providers to decide if they have the capacity and funds to invest in analytical skills training. This is likely to disadvantage the smaller sized organisations which make up the majority of homecare providers in the UK.^22^ Others described wanting to change how staff were using the digital system having observed deteriorations in the quality and depth of information recorded compared to previous paper-based systems. These areas of change both speak to the government's desired outcomes of the digitalisation of social care^4^ in terms of efficiency and person-centredness, signalling the importance of further work on these issues.

The final section of the survey explored views about the possibility of a national dataset of users of homecare services populated by anonymised data submitted by homecare providers, and the factors that may affect willingness or ability to contribute to such a dataset. Overall, there was strong support for this, though a smaller proportion reported they would definitely consider contributing to it. Concerns about costs to them in terms of staff time and data privacy were commonly held reasons behind this uncertainty. Importantly, and perhaps particularly relevant given that self-funders comprise a significant proportion of those using homecare services in the UK, client willingness was identified as a potential barrier to contributing to a national dataset by a large majority of respondents. UK research on public opinion on sharing de-identified NHS data for research purposes^30^ suggests that, whilst there is overall support, different views are held, replicating studies in other countries.^31^ However, what is not yet known in the UK context is whether how care is being funded (self- vs. local authority-funded) affects the public's views about sharing of homecare data for research purposes or for use by businesses.

## Conclusions

Findings present a mixed and dynamic situation in the digital transformation of homecare in the UK, with evidence of numerous different systems being used and homecare providers being at different stages of digitalisation. The high-pressure, low-margin context of UK homecare was observed to be exerting an influence on digitalisation. Specifically, a lack of time and financial costs were identified as constraining improvements in digital systems. Recent efforts by the UK government to increase the guidance available to homecare providers on selecting a digital care management software system may go some way to reducing the time homecare providers need to invest: it will be important to evaluate whether this is the case.

With respect to financial barriers, whilst some financial support is offered to homecare providers adopting digital systems for the first time, this is not the case for those wanting or needing to change the system(s) they are using. Yet our findings suggest a sizeable proportion of homecare providers are dissatisfied with their current digital system. We would argue consideration needs to be given to how these homecare providers can be enabled and supported to improve their digital systems. Such findings also point to the need for evaluations of the digital transformation programme to not only capture uptake of digital systems but also satisfaction with those systems. Furthermore, those with digital systems reported an under-utilisation of the data being collected, attributed to a lack of relevant analytical skills within their organisation. Unless this is addressed, the full potential of digitalisation cannot be realised. Finally, whilst there was support for a national dataset of users of homecare services, our findings suggest that a number of factors will determine whether provider decide to contribute data to it. Efforts to secure buy-in are likely to both require attention to perceived costs and addressing providers’ concerns regarding client unwillingness.

## Supplemental Material

sj-docx-1-dhj-10.1177_20552076241255477 - Supplemental material for UK homecare providers’ views about, and experiences of, digitalisation: A national survey Supplemental material, sj-docx-1-dhj-10.1177_20552076241255477 for UK homecare providers’ views about, and experiences of, digitalisation: A national survey by Jan Healey, Vanessa Davey, Jennifer Liddle, Gareth O’Rourke, Barbara Hanratty and Bryony Beresford in DIGITAL HEALTH

sj-docx-2-dhj-10.1177_20552076241255477 - Supplemental material for UK homecare providers’ views about, and experiences of, digitalisation: A national survey Supplemental material, sj-docx-2-dhj-10.1177_20552076241255477 for UK homecare providers’ views about, and experiences of, digitalisation: A national survey by Jan Healey, Vanessa Davey, Jennifer Liddle, Gareth O’Rourke, Barbara Hanratty and Bryony Beresford in DIGITAL HEALTH
