# Supplementary material for: UK homecare providers’ views about, and experiences of, digitalisation: A national survey
Source: Digit Health. 2024 May 22;10:20552076241255477. doi: 10.1177/20552076241255477 (PMC11113022; doi:10.1177/20552076241255477)
Supplement: sj-docx-1-dhj-10.1177_20552076241255477 - Supplemental material for UK homecare providers’ views about, and experiences of, digitalisation: A national survey [file sj-docx-1-dhj-10.1177_20552076241255477.docx]

**FRONT PAGE**

**The DACHA-DOM survey of UK homecare providers**

**Thank you very much for taking the time to complete this survey on behalf of your homecare organisation/service.**

**About taking part in the DACHA-DOM survey**

The survey should take no more than 10-15 minutes to complete. It’s fine to do this over more than one session, but you will need to access it from the same device, and using the same browser.

The progress bar at the top of your screen shows progress through the survey.

You can take part anonymously: you do not have to provide any information about yourself or the organisation you are reporting on.

*Please note: at the end of the survey you have the option to identify the homecare organisation you are representing. You do not have to answer this question. We will only use this information to broadly describe the types of homecare services/organisations taking part in the survey.*

The survey closes on [*Wednesday, 18 November*].

Further information about how we will use and look after the data this survey collects, please refer to our Information Sheet [*hyperlinked*].

If you have any questions, please contact the DACHA-DOM research team: [*researcher email*]

# **SECTION 1: ABOUT YOUR HOMECARE ORGANISATION/SERVICE**

This section asks about you and the homecare organisation/service you are reporting on.

**1.1 What is your role within your homecare organisation?**

1. Business owner / Franchisee
2. Chief Executive Officer/Executive Director/Director
3. Senior Manager
4. Registered Manager
5. Other (*please state*)

**1.2 Which statement best describes the type of organisation/service you’ll be reporting in for this survey.**

1. Franchise run independently but in accordance with the franchisor branding and standards
2. Local office/branch of a chain business
3. Independent business
4. Local authority in-house service
5. Chain central/national office
6. Other (*please state*)

**1.3 [If 1.2=a] Will you be filling in this survey about one or more franchises?**

1. One franchise
2. More than one franchise. Please state how many: [*FREE TEXT*]

**1.4 [If 1.2=c,e,f) Please tell us about the size of your homecare service/organisation**

1. 1 office/operating base
2. 2 - 4 offices (or branches)
3. 5 - 10 offices
4. 11 - 19 offices
5. 20+ offices

**1.5 [If 1.2=d not selected] Which statement best describes your homecare organisation/service?**

1. For-profit
2. Not for profit
3. Social Enterprise
4. Other (*please state*)

PAGE BREAK

**1.6 Which type(s) of homecare does your organisation provide?** (*Please tick all that apply*)

1. Regular domiciliary care visits
2. Reablement
3. Live-in care
4. Continuing Health Care (CHC) funded package of care
5. Other (please state)

**1.7 Does your organisation also provide either of the following?** *(Please tick all that apply)*

1. Care home or nursing home
2. Assisted living/extra care housing
3. Neither of the above

**1.8 Is your homecare organisation/service TTDI (Treatment of Disease, Disorder and Injury) registered?**

a. Yes

b. No

c. Unsure

PAGE BREAK

**1.9 In which UK country/ies do you operate? (*Please tick all which apply)***

1. England
2. Wales
3. Scotland
4. Northern Ireland

**1.10 [Only If 1.9=a] Which geographical regions(s) do you operate in? (*Please tick all which apply*)**

- [*Nine English regions listed: multiple choice*]

**1.11 Approximately how many clients is your organisation/service currently providing homecare to?**

1. [*FREE TEXT*]

**1.12 [Only if 1.2=a,b,c,e,f,g ] Which statement best describes your clients?**

1. All or mostly local authority or NHS-funded (including direct payments)
2. All or mostly self-funded
3. Roughly equal numbers of LA/NHS funded and self-funded
4. Other (*please state*)

**1.13 Does your organisation submit information to the ‘adult social care worforce’ dataset via the Skills for Care platform?**

1. Yes
2. No
3. Unsure

**SECTION 2: SYSTEMS USED TO COLLECT AND STORE INFORMATION**

This section asks about the systems your organisation uses to collect and store information about its clients.

**2.1 Overall, how would you describe the way your organisation records and stores information about its clients and their care?**

1. All or predominantly paper-based
2. Mix of paper records and digital files/software
3. All or predominantly digital files/software

**2.2 What types of information do you routinely have available in digital format for all your clients: either because it is recorded digitally, or because you subsequently input it into digital files/software?** *(Tick all that apply)*

1. Basic information about the client (e.g. age, gender, size of household, NHS number)
2. Care needs assessment information
3. Care package details
4. Daily care visit details
5. Client satisfaction surveys
6. Other – please state

**2.3 Please tell us the main software systems your homecare organisation uses.**

[FREE TEXT]

**2.4 Do any/all of these systems allow you to run ‘queries’ and/or database searches?**

1. Yes
2. No
3. Unsure

**2.5 How does your homecare organisation use the digital data it has about its clients and their care?** (*Tick all that apply*)

1. Understand/monitor the needs profiles of clients
2. Monitor client experience / satisfaction
3. Monitor client outcomes
4. Inform staffing/workforce planning
5. Identify staff training needs
6. Inform business development decisions
7. Other (*please specify*)
8. None of the above

**PAGE BREAK**

**2.6** **Do you provide your homecare workers with a mobile device (e.g. phone, tablet) or laptop?**

1. Yes, all homecare workers
2. Yes, senior homecare workers only
3. No
4. Other – please state

**2.7** **[IF Q2.6=a, b or d]** **What type(s) of device do you provide?** (*Tick all that apply)*

1. Mobile phone
2. Tablet
3. Laptop

**2.8 [IF Q2.6=a, b or d] Please tell the reasons why your organisations provide homecare workers with a mobile device.** (*Tick all that apply*)

1. Scheduling
2. Time tracking
3. Completing daily care records
4. Supporting communication with office staff
5. Supporting communication between homecare staff
6. Other – please state
7. None of the above

**2.9** **Do mobile connectivity issues in clients’ homes, or the wider locality, impact how you use mobile devices?**

a. Yes – please state

b. No

**SECTION 3: VIEWS ABOUT YOUR CURRENT INFORMATION SYSTEMS**

This section is about any changes you’d like to make to the systems you use to collect and store information, and any barriers to implementing these changes.

- 1. **Are there things you’d like to change or improve in the way your organisation currently collects, records and/or stores information about its clients and their care ?**

1. Yes
2. No

**3.2** [*If 3.1=Yes*] **Please briefly tell us the sorts of changes or improvements you’d like to make.**

[*FREE TEXT*]

**3.3** [*If 3.1=Yes*]: **What are the main things getting in the way, or which make it difficult, to implement these changes or improvements?**

[*FREE TEXT*]

# **SECTION 4: THE DIFFERENT TYPES OF INFORMATION YOU COLLECT**

This section asks about the different types of information you routinely collect about your clients and when it might be updated. By routinely we mean you would expect to have this information for all your clients.

**4.1 To start, please tell us how often clients’ care packages are usually reviewed.**

1. Annually
2. Every 6 months
3. Other – (please state)

**4.2 Now turning to when someone first starts using your service, which of the following do you routinely collect?** *(Tick all that apply)*

1. Who made the referral (e.g. client/family, local authority)
2. Who is funding the care package (e.g. self-funded, direct payment, local authority, NHS)
3. NHS number
4. Local Authority Reference Number
5. National Insurance number

**4.3 In terms of information about health/disabilities, which of the following do routinely collect when someone first starts using your service?** *(Tick all that apply)*

1. Diagnosed physical health condition(s)
2. Diagnosed mental health condition(s)
3. Memory or cognitive impairment
4. Sight or hearing impairment(s)
5. Communication needs
6. Diagnosed learning disabilities
7. Autism diagnosis

**4.4 When would this information be updated (e.g. new diagnoses, changes in existing health conditions/impairments)? (*You can tick more than one response for each item*.)**

|  | When changes occur | At care package reviews | No standard practice | Not updated |
| --- | --- | --- | --- | --- |
| Populated from 4.3 |  |  |  |  |

**PAGE BREAK**

**4.5 When someone first starts using your service, do you routinely collect information about the involvement of other services and informal carers?** (Tick all that apply)

1. Other social care services
2. Primary care/community health care
3. Hospital clinics/services involved
4. Informal carers

**4.6. When would this information be updated? (*You can tick more than one response for each item*.)**

|  | When changes occur | At care package reviews | No standard practice | Not updated |
| --- | --- | --- | --- | --- |
| Populated from 4.5 |  |  |  |  |

**4.7 Which of the following are routinely recorded in a client’s daily care file/record (paper or digital)?** (*Tick all that apply*)

1. Care tasks completed
2. Medication prompting and administered (MAR chart)
3. Any healthcare tasks performed (e.g. wound care, health status monitoring).
4. Adverse events or incidents
5. Observed changes in a client’s health or well-being (e.g. pain, confusion, mood)

**4.8 When is information about care needs, goals and preferences, and care package details updated in your records? (*You can tick more than one response for each item*.)**

|  | When changes occur | At care package reviews | No standard practice | Not  updated |
| --- | --- | --- | --- | --- |
| Care needs (e.g. personal care, domestic, mobility, medication) |  |  |  |  |
| Regular medication(s) |  |  |  |  |
| Client/family desired care outcomes/goals |  |  |  |  |
| Preferences for how care is delivered |  |  |  |  |
| Care package details (e.g care tasks/activities, number/duration of visits, times of day etc.) |  |  |  |  |

- 1. **What types of information are routinely recorded when a client stops using your service?** (*Tick all that apply*)

1. Reason why stopped using service
2. Information about next care destination/service
3. Other – please state
4. We don’t routinely record this type of information

# **SECTION 5: USE OF STANDARD QUESTIONNAIRES & ASSESSMENT TOOLS**

In this penultimate section we ask about any questionnaires or tools you use to measure client experience and outcomes. We also ask about assessment tools you may use to monitor aspects of a client’s health or functioning.

**5.1 Does your organisation use a client satisfaction survey/questionnaire (or similar)?**

1. Yes, we use one we developed ourselves
2. Yes, we use one developed elsewhere.
3. No

**5.2 Does your organisation routinely use any of these quality of life measures for assessment or monitoring purposes?** *(Tick all that apply)*

1. Adult Social Care Outcomes Tool (ASCOT)
2. ICECAP Quality of life measure
3. Euro-QoL EQ-5D or EQ 3-D
4. R-outcomes
5. Other (*Please state*)
6. None of the above

**5.3 Would your organisation ever use a standard assessment tool for any of the following? (This *excludes* instances when another service asks you to do so).** (*Tick all which apply*)

1. Independence/ functioning (e.g. Barthel Index)
2. Sleep (e.g. Sleep Quality Scale)
3. Pain (e.g. Abbey Pain Scale)
4. Skin Condition (e.g. Waterlow scale)
5. Mood (e.g. Geriatric Depression Scale)
6. Frailty (e.g. Clinical Frailty Scale)
7. Physical Activity (e.g. Physical activity Scale for the Elderly (PASE))
8. Loneliness (e.g. UCLA Loneliness Scale)
9. Social Networks (e.g. Lubben Social Networks Scale)
10. Nutrition (e.g. MUST screening tool)
11. None of the above

# **SECTION 6: YOUR VIEWS ABOUT A NATIONAL DATASET FOR HOMECARE**

Finally, we’d like to ask about your views on whether creating a national dataset for homecare is needed, and would be feasible to achieve.

**6.1 At a national level we know very little about the population of older people who use homecare. A voluntary national dataset on homecare users (using non-identifiable data submitted by homecare providers) could be a solution. Do you think, in principle, this is a good idea?**

1. Yes
2. No
3. Unsure

**6.2 Would your homecare service/organisation consider contributing to such a dataset?** *Please tick the statement which best describes how you feel about this.*

1. Yes
2. Maybe
3. No
4. Don’t know

**6.3 Which of the following might affect your willingness or ability to submit data to a national dataset on homecare users.** *(Tick all that apply)*

1. Concerns around client privacy/General Data Protection Regulations (GDPR)
2. Client willingness for their anonymised data to be shared outside your homecare service/organisation
3. Staff time costs
4. Software costs
5. Hardware costs (e.g. computers, mobile phones)
6. Insufficient data management/technology skills within management/admin team
7. Some/all data stored in paper format
8. Data stored in multiple formats/systems
9. Commercial sensitivities
10. Other. (*Please specify*)

**6.4 The next stage of this research involves the research team meeting with homecare providers (and other stakeholders) to further explore the idea of a minimum dataset for homecare. Might you/your organisation be interested in taking part in this?**

1. Yes
2. No

**6.5.Would you like to receive a summary of the findings from this survey?**

1. Yes
2. No

**6.6** [*If yes to 6.4 or 6.5*]: **Please provide your contact details. Please be assured, this information will only be used to contact you as requested. It will be stored separately to your survey responses.**

- Name: [*FREE TEXT*]
- Email: [*FREE TEXT*]

**6.7 Before submitting your survey you have the option of telling us the name of the homecare service/organisation you work for. You do not have to do this. This information will not be reported or shared. We will only use this information to broadly describe the types of homecare services/organisations who take part in this survey.**

[FREE TEXT]

[SUBMIT]
